# Effective Enrichment of Plasmonic Hotspots for SERS by Spinning Droplets on a Slippery Concave Dome Array

**DOI:** 10.3390/bios12050270

**Published:** 2022-04-24

**Authors:** Jialin Wu, Jianpeng Cai, Yuan Fan, Ying Zhang, Hui Fang, Sheng Yan

**Affiliations:** 1 Nanophotonics Research Center, Institute of Microscale Optoelectronics, Shenzhen University, Shenzhen 518060, China; 2070496010@email.szu.edu.cn (J.W.); 1900453026@email.szu.edu.cn (J.C.); 2176285320@email.szu.edu.cn (Y.F.); 1950453026@email.szu.edu.cn (Y.Z.); 2 College of Physics and Optoelectronics Engineering, Shenzhen University, Shenzhen 518060, China; 3 Institute for Advanced Study, Shenzhen University, Shenzhen 518060, China

**Keywords:** droplet manipulation, SERS, coffee-ring effect, biosensing

## Abstract

Surface-enhanced Raman scattering (SERS) detection requires dense hotspots and a uniform distribution of analytes to obtain a stable signal with good repeatability. However, due to the coffee-ring effect on the hydrophilic substrate, and the difficulty of droplet manipulation on the superhydrophobic substrate, few substrates can ensure that the analytes are evenly distributed. In this work, we develop a method that can efficiently enrich plasmonic hotspots for SERS measurement on the superhydrophobic concave dome array (SCDA). The SCDA is formed by spraying hydrophobic silica nanoparticles onto a polydimethylsiloxane (PDMS) slab with a concave dome array that can physically confine the droplets and overcome the coffee-ring effect. During droplet evaporation, the SCDA is driven by a horizontal spinner, and the droplets spin on the SCDA, enabling the plasmonic nanoparticles to become closely packed to form the SERS hotspots. The limit of detection (LOD) of the dynamic-enriched SERS hotspots for crystal violet and methylene blue can reach up to 10^−11^ M. Moreover, the LOD for melamine in milk can reach 5 × 10^−7^ M, which is lower than the safety threshold defined by the Food and Drug Administration (FDA). Based on this SERS platform, an effective, low-cost, and simple method for SERS detection in analytical chemistry and food safety is highly expected.

## 1. Introduction

When a droplet of suspension situated on a hydrophilic surface with a low contact angle evaporates, its suspended matter will predominately deposit at the outer boundary; this process is called the coffee-ring effect [1]. During evaporation, the droplet features not only an air–water–solid interface, forming a spherical cap, but also an internal Marangoni flow due to small temperature gradients, thus bringing the suspended matter (which is usually composed of small particles) to the outer ring [2]. A natural consequence is that the suspended particles will be highly concentrated along the edge of the original droplet, but loosely distributed at the center. The coffee-ring effect occurs in a wide variety of particles, ranging from large colloids [3,4] to nanoparticles [5]. In many applications based on sample preparation through droplet evaporation, the uneven particle distribution will affect the quality of the measurement. For example, in surface-enhanced Raman scattering (SERS) detection, strong uniformity of the Raman signal occurs [6].

One of the effective approaches to suppress the coffee-ring effect is to replace the hydrophilic surface with a superhydrophobic surface on which a droplet with a high contact angle will form [7]. With the advancement of fabrication technologies, such as photolithography [8], e-beam lithography [9], and stereolithography [10], some nature-inspired superhydrophobic micro/nanostructures have been developed for SERS detection [11,12]. However, these technologies rely on bulky and expensive facilities, which somewhat limits their accessibility. As an alternative approach, electrochemical deposition can deposit metal nanostructures onto a specific substrate to create superhydrophobic surface [13]. In addition, the SERS substrate with plasmonic nanostructures created using the electrochemical deposition method has high stability and enhances SERS signal [14]. The order of the plasmonic nanostructures is highly dependent of the precise control of the chemical reaction.

Several smart methods used to prepare the superhydrophobic surfaces are reported that can enrich the mixture of the plasmonic nanoparticles and analytes. For example, a nanotexture surface infiltrated with a perfluorinated liquid to form the slippery liquid-infused porous surface (SLIPS) can concentrate the analytes and plasmonic particles for attomole-level detection [15]. Any disturbance will cause the droplets to slip away from the surface due to the superhydrophobicity. Therefore, a superhydrophobic and magnetically functionalized surface was developed by spraying silica nanoparticles onto the surface of a magnetorheological elastomer, where several droplets can steadily stand in the magnetically-induced deformation area [16,17]. Unfortunately, the functionalized surface is unable to process abundant droplets simultaneously due to the interference of the magnets. Therefore, it is still challenging to develop a facile and efficient way to achieve multi-droplet manipulation to prepare the scalable SERS hotspots for multiplexing detection.

Here, we propose a superhydrophobic concave dome array (SCDA) for the dynamic enrichment of plasmonic nanoparticles. Each droplet with plasmonic nanoparticles can stay in each concave dome during the spontaneous evaporative process to generate the SERS hotspots. Unlike the plain superhydrophobic surface that allows droplets to slip from the substrate, the concave dome can physically confine the droplets. Therefore, multiple SERS hotspots can be prepared simultaneously on the same substrate. Similar to the commercial multi-well Petri dish, this SERS array is able to realize multi-concentration and multi-target detection.

## 2. Materials and Methodology

### 2.1. Preparation of SCDA

**Figure 1:**
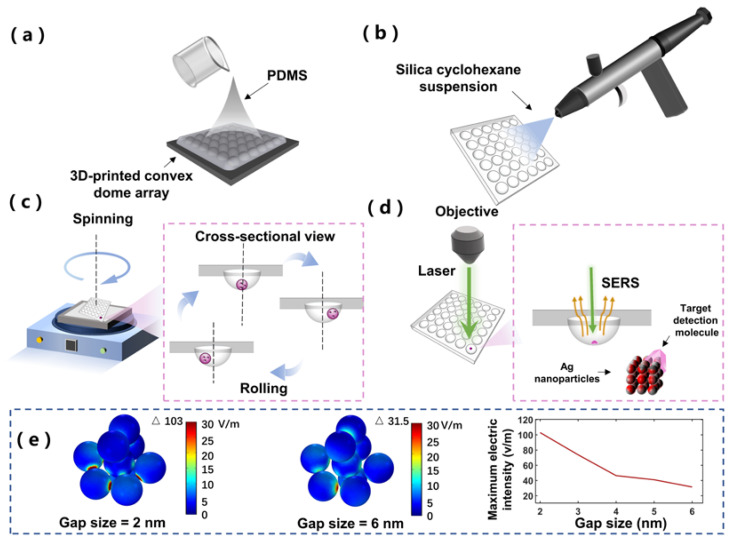
(**a**) Schematic diagram of concave dome array preparation; (**b**) schematic diagram of superhydrophobic surface coating; (**c**) schematic diagram of working mechanism of dynamic enrichment on SCDA; (**d**) schematic diagram of surface-enhanced Raman scattering (SERS) detection on SCDA; (**e**) numerical simulation of electric field intensity of Ag nanoparticles with different gap sizes in three-dimensional space.

Figure 1a,b describes the preparation process of SCDA. First, the polydimethylsiloxane (PDMS) concave dome array was replicated from the 3D-printed convex dome array, as shown in Figure 1a. Specifically, a mold with an overall size of 50 mm × 50 mm and a 6 × 6 convex dome array on the surface was printed with a 3D printer. The convex dome was designed as a semi-sphere with a radius of 3.5 mm and a distance between domes of 8 mm. The printed mold will be treated with the release agent. The PDMS gel and curing agent were mixed evenly with the mass ratio of 10:1. After the bubbles in the mixed gel were removed under vacuum, the mixed gel was poured onto the 3D-printed convex dome array. To avoid the deformation of 3D-printed mold, the mixture gel was cured at 50 °C for 10 h. After the PDMS was fully cured, a concave dome array was formed after peeling off.

Next, a superhydrophobic surface was prepared by spraying silica nanoparticle suspension, as shown in Figure 1b. The silica nanoparticle suspension was prepared by mixing 0.8 g hydrophobic nanosilica, 0.45 g PDMS mixture, and 40 mL cyclohexane, and was then sealed to avoid cyclohexane evaporation. To avoid the agglomeration of the nanosilica, the silica nanoparticle suspension was put into the ultrasonic oscillator for 30 min (frequency is 40 kHz). Significantly, the temperature should be kept below 30 °C to avoid partial solidification of PDMS in the solution. Subsequently, the suspension was evenly mixed by magnetic stirring for 30 min (at a speed of 700 revolutions per minute (RPM)). After complete mixing, 10 mL mixed suspension was transferred into an airbrush cup, and then the suspension was evenly sprayed onto the surface of the concave PDMS substrate. During spraying, the airbrush was connected to the air compressor under a constant pressure of 4 bar, and the distance between the nozzle (diameter of 0.3 mm) and the concave dome array was kept at 15 cm. The working principle of suspension is that cyclohexane enables the concave dome array to swell, and hydrophobic silica nanoparticles adhere to the swollen surface; PDMS allows the hydrophobic silica nanoparticles to firmly adhere to the surface of concave dome array. After spraying, the concave dome array was horizontally placed on a hotplate at 150 °C for 40 min to enhance the evaporation of cyclohexane and cure the PDMS. The excess silica nanoparticles on the SCDA surface were rinsed with distilled (DI) water to obtain a clean substrate.

### 2.2. Working Mechanism

Figure 1c shows the working principle of enriching plasmonic nanoparticles and analytes by rotating the SCDA. Because of the properties of the high static water contact angle and concave dome structure of the SCDA, droplets can easily roll in the concave dome, but will not leave the substrate due to the concave structure (Videos S1 and S2). When a droplet is dropped into one of the wells of the SCDA, the plasmonic nanoparticles in the droplet can be fully and evenly mixed with the analytes through periodic rotation (Video S3). During the evaporation process, nanoparticles follow the movement of the droplets, and are not easy to deposit at the contact line between the droplet and the substrate so as to effectively suppress the coffee-ring effect. [18]. Therefore, the nanoparticles can be concentrated into a small area to enhance the Raman signal. As the droplet volume gradually decreases during the evaporative process (Figure S1), the microdroplets begin to adhere to SCDA, and cannot rotate freely and dynamically. At this time, three-dimensional SERS hotspots will be formed inside the microdroplet. At the same time, the analytes are actively captured in small gaps (hotspots) to further improve the Raman signal, as shown in Figure 1d [19,20].

### 2.3. Materials

PDMS gel and curing agent were purchased from Dow Corning (Midland, USA). Hydrophobic silica nanoparticles with an average size of 250 nm were purchased from Macklin (Shanghai, China), and used to make superhydrophobic coating suspension. The Ag colloid was from Beijing Biotyscience company (Beijing, China), with a diameter of 60 nm and concentration of 0.1 mg/mL. Crystal violet (CV), methylene blue (MB), and melamine were all purchased from Macklin (Shanghai, China) to test the SERS detection on the SCDA.

### 2.4. Characterization and Experimental Setup

The images of enriched nanoparticles, after the evaporation of droplets, on different substrates and different enrichment modes were analyzed using a high-resolution field emission scanning electron microscope (SEM) (ZEISS SUPRA^®^ 55, Carl Zeiss, Oberkochen, Germany). CA values were obtained with an optical contact angle measuring instrument (Theta, Biolin scientific, Gothenburg, Sweden). An optical magnifier (HAG0950, SHOCREX, Shenzhen, China) was used to record the evaporative process of the droplets and observe the laser point. A horizontal rotator (NSP-300, NuoMi, Suzhou, China) provided the periodic rotation for droplet spinning on the SCDA.

The Raman signal detection system included a Raman spectrometer (iHR550, Horiba, Kyoto, Japan), two long-pass filters (RL-532nm, Shanghai-optics, Nanjing, China), a laser (532 nm, Ventus, Konstanz, Germany), a lens with a focal length of 2 mm, and an objective lens of 20× magnification and numerical aperture (NA) of 0.4. The diameter of detection spot was about 1.6 μm. The exposure time was 5 s, and the laser power was about 12 mW.

As shown in Figure S2, the side view is similar to the diagram in Figure 1d. The SCDA was placed under the objective lens of 20× magnification. The top view illustrates the collection optical path of the Raman signal. A filter was placed in the collection optical path to filter Rayleigh light, and then a 10× objective lens (NA is 0.3) was used to focus the Raman signal to the optical fiber port to collect the Raman signal directly.

### 2.5. Numerical Simulation for SERS

To verify that evaporation of droplets on SCDA can form effective SERS hotspots, the wave optical module of COMSOL was used for numerical simulation of electric field distribution of Ag particle aggregation, with the particle gap either at 2 nm or 6 nm. Figure 1e shows the electric field intensity of such configurations, with randomly distributed Ag particles in three-dimensional space, where nine Ag particles all with a diameter of 60 nm are illuminated by the 532 nm optical plane wave. In the simulation model, a sphere domain with a diameter of 400 nm was set as the water, and an outer layer of 800 nm thickness was set as the perfect match layer (PML).

## 3. Results and Discussion

### 3.1. Investigation of SCDA

**Figure 2:**
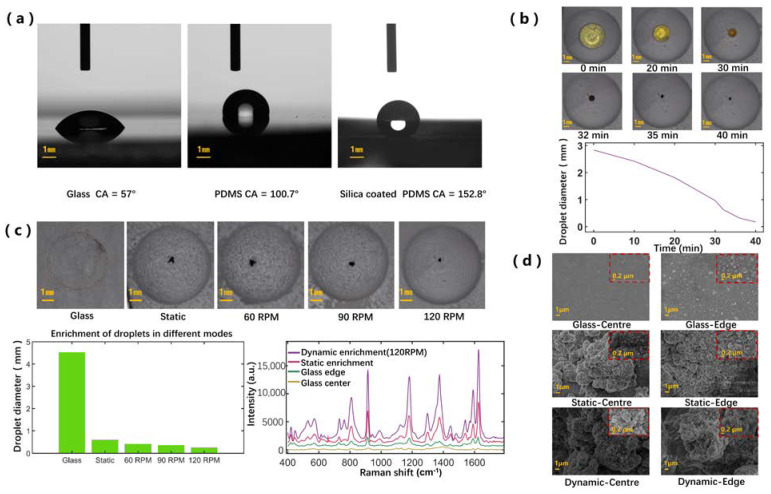
(**a**) Optical images of the 10 μL pure water droplet on different substrates showing different contact angles; (**b**) optical images and time-dependent curve showing the volume change of the droplet, containing 13 μL droplet during rotary evaporation at a spinning speed of 120 RPM; (**c**) optical images, related histogram, and SERS signals for the evaporation-ended droplet on glass and on SCDA spun at different speeds; (**d**) SEM images of droplets vaporized on different substrates and at different locations.

To verify the superhydrophobicity of SCDA, we measured the droplet contact angle on the plane substrate coated with the silica suspension, and compared with contact angle on the glass surface and PDMS surface. Since our optical contact angle measuring instrument cannot measure the contact angle in the concave dome, the data for the contact angle on the superhydrophobic concave dome are not available. Figure 2a shows an optical image of 10 μL pure water on these three types of surfaces. The superhydrophobic surface shows the average contact angle of 152.8°, the PDMS surface 100.7°, and the glass surface 57° (all from multiple measurements).

To further study the enrichment capability during droplet dynamic evaporation on the SCDA, we recorded the optical images displaying the diameter change of the droplets (13 μL), as shown in Figure 2b. The SCDA was spun at a speed of 120 RPM. To speed up the evaporation, we illuminated the SCDA with an incandescent lamp. Over time, the volume of the droplet gradually decreased; finally, the droplet was concentrated into a small area of about 0.17 mm in diameter.

### 3.2. Comparison of the Deposition Patterns and SERS Performance Based on Different Enrichment Modes

To study the deposition property and SERS performance of droplets under different spinning speeds applied to the SCDA, we consistently started with the droplet composed of 10 μL CV solution (10^−8^ M) and 3 μL Ag colloid. As shown in Figure 2c, the smallest enrichment area can be reached when the spinning speed is set as high as 120 RPM. This phenomenon may be attributed to the fact that the higher spinning speed reduces the contact time between the moving droplet and the substrate, thus effectively reducing the droplet adhesion with the substrate surface [21]. Previous studies found that there is a vortex zone above the contact line for stationary evaporating droplets, where most of the nanoparticles are deposited [18]. For moving droplets, there is a vortex zone that accounts for most of the droplets. The interior nanoparticles rotate with the vortex zone, so the nanoparticles are more concentrated and enriched on the substrate during the evaporative process. In the experiment, when the spinning speed exceeded 120 RPM, the area of the final deposition of the droplet was not further reduced. The reason for this may be that when the speed exceeds 120 RPM, the droplets do not follow the movement of the SCDA, and are unable to rotate synchronously with the SCDA. Therefore, the higher speed cannot further concentrate the plasmonic nanoparticles into a smaller area. The spinning speed of 120 RPM was therefore used here. Furthermore, as expected, the coffee-ring effect was evident when the droplets evaporated on the glass sheet.

In the next step, the SERS measurements were performed on the prepared samples due to the dynamic enrichment processes of the droplets. In Figure 2c, the SERS signals corresponding to the droplets undergoing different enrichment processes are presented. The SERS signal for the dynamic enrichment on the SCDA was the strongest, followed by, several times lower, the signal for the static enrichment on the SCDA; those signals measured from the glass plate (one from center and the other from the edge) were both much lower. The CV molecules usually show the typical Raman peaks at 807 cm^−1^ and 915 cm^−1^ (both from the symmetric stretch of the dimethylamino bond C-N-C), 1182 cm^−1^ (from the stretching vibration of C-N), 1373 cm^−1^ (from the stretching vibration of C=C), and 1624 cm^−1^ (from the stretching vibration of C=C) [22]. Here, we quantitatively studied the Raman peak at 915 cm^−1^, and straightforward calculation revealed the following: the SERS signal for the SCDA dynamic enrichment was 2.2 times that for the SCDA static enrichment, 7.7 times that for the edge of the glass sheet, and 39.7 times that for the center of the glass sheet.

We further continued on the estimation of the Raman enhancement factor for the SCDA dynamic enrichment case, based on the following formula [23]:

(1) EF=ISERSCSERSIRamanCRaman

where $I_{SERS}$ and $I_{Raman}$ represent the Raman intensity, respectively, for the SERS measurement and the conventional Raman measurement (without Ag nanoparticles), and $C_{SERS}$ and $C_{Raman}$, respectively, represent the corresponding solution concentrations of analyte by plugging in the values $C_{SERS}$ = 10^−8^ M and $C_{Raman}$ = 10^−3^ M (refer to Figure S3); we subsequently obtained EF = 3.55 × 10^5^. 

As the final step, we took the SEM images for all the above samples, as shown in Figure 2d. Each of the large images were taken at the 5000 magnification, while each inlet small images were taken at 30,000 magnification. For the glass sheet, there were obviously more Ag nanoparticles at the edge compared to the center; for the static enrichment case, the Ag nanoparticles were still denser at the edge, but the contrast was much weaker. Finally, for the dynamic enrichment case, the Ag nanoparticles at both the edge and the center appear closely packed, indicating the resulting even distribution of the Ag nanoparticles.

### 3.3. SERS Performance Study of the SCDA

**Figure 3:**
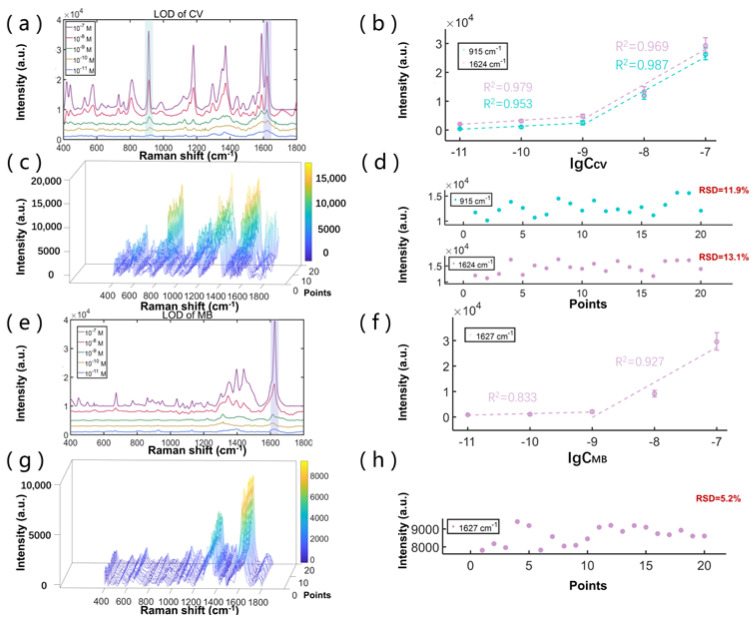
(**a**) SERS signals from dynamically enriching 13 μL droplet (10 μL CV solution and 3 μL Ag colloid) with different concentrations; (**b**) the data for SERS intensity peaks at 915 cm^−1^ and 1624 cm^−1^ versus the CV concentration; (**c**) SERS signals of 10^−8^ M CV/AgNPs from 20 random positions based on SCDA dynamic enrichment mode; (**d**) the data for CV SERS intensity at 915 cm^−1^ and 1624 cm^−1^ from 20 random positions; (**e**) SERS signals from dynamically enriching 13 μL droplet (10 μL MB solution and 3 μL Ag colloid) with different concentrations; (**f**) the data for SERS intensity peaks at 1627 cm^−1^ versus the MB concentration; (**g**) SERS signals of 10^−8^ M MB/AgNPs from 20 random positions based on SCDA dynamic enrichment mode; (**h**) the data for MB SERS intensity at 1627 cm^−1^ from 20 random positions.

To further study the limit of detection (LOD) and the signal uniformity of the dynamic enrichment mode of SCDA, we used CV and MB, respectively, as the probe molecules to prepare multiple detection hotspots with different concentrations of these two molecules on the same SCDA. Figure 3a,e shows the SERS signals for different molar concentrations, ranging from 10^−7^ M to 10^−11^ M, while Figure 3c,g shows the SERS signals for 20 randomly chosen positions when the molar concentration is at 10^−8^ M. 

For the CV molecules, several Raman characteristic peaks can still be discerned even for concentrations as low as 10^−11^ M (Figure 3a). In Figure 3b, we further plotted the data of the Raman peak intensity versus the molecular concentration for the Raman peaks at 915 cm^−1^ and 1624 cm^−1^, respectively. Apparently, the intensity points can be separated into two linearly fitting regions, and at the higher concentration region, the slope is much larger. The resulting linear fits for the 915 cm^−1^ peak are as follows: for the higher concentration region, Y_915cm_^−1^ = 11823·lgC_CV_ + 108157, and for the lower concentration region, Y_915cm_^−1^ = 1078·lgC_CV_ + 12138, with correlation coefficients (R^2^) of 0.987 and 0.953, respectively. These results imply that SCDA is suitable for quantitative analysis at high concentrations, and qualitative analysis at low concentrations.

The 20 random points of the SERS signals show similar spectral structures and signal strengths, demonstrating the signal uniformity of our method (Figure 3c). The detailed examination of the intensity points for the Raman peak at 915 cm^−1^ and at 1624 cm^−1^ are plotted in Figure 3d. The statistical calculation conducted on these intensity points revealed that the relative standard deviations (RSD) are 11.9% and 13.1%, respectively, showing high signal uniformity. The slight difference in Raman signals may be attributed to the slight vibration of the experimental table, and the pulse type of the laser.

We further performed the exact same measurements on the MB molecules. In Figure 3e, the SERS signals show the MB characteristic peaks at 1397 cm^−1^ (from the asymmetric C–N stretching) and 1627 cm^−1^ (from the C–C ring stretching) [24]. As shown in Figure 3f, the linear fits for the 1627 cm^−1^ appear as Y_1627cm_^−1^ = 13,735·lgC_MB_ + 123,467 (R^2^ equals to 0.927) for the higher concentration region, and Y_1627cm_^−1^ = 597.45·lgC_MB_ + 7324 (R^2^ equals to 0.833) for the lower concentration region. As presented in Figure 3g–h, the RSD of the SERS intensity at 1627 cm^−1^ for the 20 measuring points was calculated to be 5.2%, which was attributed to the relatively uniform distribution of the CV molecules and Ag colloids. The experimental results show that the dynamic enrichment mode can not only concentrate the analytes and plasmonic nanoparticles into a smaller area to obtain dense hotspots for better SERS measurement, but it can also obtain a uniform signal with good repeatability.

As shown in Table 1, compared with other substrates, SCDA has the ability to manipulate abundant droplets and make multiple hotspots at the same time. Within the same time period, where other methods can only prepare one droplet, our method can prepare numerous droplets. Therefore, when a large number of hotspots is demanded, SCDA is more applicable. Moreover, the LOD of our method can be improved by using an objective lens with a higher numerical aperture for collecting the intensified Raman signals.

### 3.4. SERS Detection of Melamine

Melamine is a triazine heterocyclic organic compound that is widely used in the production of melamine resin, flame retardants, fertilizers, and other products. Due to its high nitrogen content (66% mass nitrogen), melamine is sometimes illegally added to the dairy products to increase their apparent protein content [26]. To prohibit such a problem, the Food and Drug Administration (FDA) has set a safe threshold of 1 part per million (ppm) (8 × 10^−6^ M) for melamine intake in infant formula, and 2.5 ppm for food and dairy products [27].

**Figure 4:**
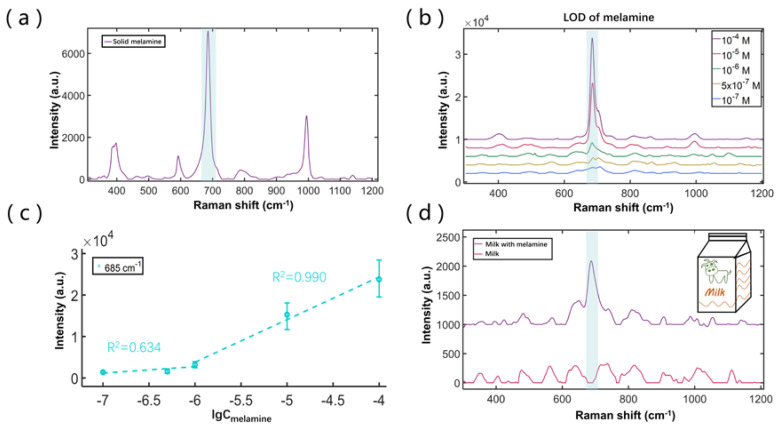
(**a**) Raman spectrum of solid melamine measured on the glass sheet; (**b**) SERS signals from dynamically enriching 13 μL droplet (10 μL melamine solution and 3 μL Ag colloid) with different concentrations; (**c**) data for SERS intensity peaks at 685 cm^−1^ versus melamine concentration; (**d**) Raman spectrum of pure milk and SERS signal of contaminated milk with melamine.

To verify the feasibility of SCDA application in the field of food safety, we performed the SERS study by mixing melamine into milk as the sample. In a controlled experiment, the Raman spectrum of solid melamine was first measured by ourselves (Figure 4a) to identify the characteristic peaks of 685 cm^−1^ (associated with ring breathing mode II of the in-plane triazine ring). We then measured the SERS signals of melamine solutions at the various concentrations, as shown in Figure 4b,c. The results show that the LOD is about 5 × 10^−7^ M. From Figure 4c, the intensity points can also be separated into two linearly fitting regions, with the higher concentration region as Y_685cm_^−1^ = 10281·lgC_melamine_ + 65484, and the lower concentration region as Y_685cm_^−1^ = 1546·lgC_melamine_ + 12009. The results imply that the SCDA can be used for quantitative analysis when the concentration of melamine is higher than 10^−6^ M.

We then prepared the milk sample by mixing melamine at a concentration of 8 × 10^−6^ M, which is just within the safety threshold. As shown in Figure 4d, the Raman spectrum of the pure milk (the red curve) does not display the 685 cm^−1^ peak. In contrast, the Raman spectrum of the mixture (the violet curve) displays a pronounced peak at 685 cm^−1^, indicating a strong capability for practical melamine detection. 

## 4. Conclusions

In this work, we developed a superhydrophobic concave dome array (SCDA) for the dynamic enrichment of plasmonic nanoparticles that can achieve the uniform SERS signal measurement. Due to low surface adhesion, the SCDA can effectively suppress the coffee-ring effect by dynamically enriching the droplets. A droplet with the initial volume of 13 μL can be condensed into a small region of about 0.17 mm in diameter after the complete evaporation of the solvent. The LOD of CV and MB molecules can reach up to 10^−11^ M. As a proof-of-concept application, these dynamic-enriched SERS hotspots can detect the melamine in milk at concentrations lower than the FDA standard. We expect our method have more potential applications in analytical chemistry and biomedicine. 

## Figures and Tables

**Table 1:** Comparing performance of SERS substrates with other reported methods.

Method	Analyte	Time	LOD	Droplet Manipulation	Number of Droplets Prepared Simultaneously	Ref.
Light-trapping SERS substrate	R6G	Not available	10^−13^ M	Not applicable	1	[12]
Taro-leaf@Ag	R6G	120 min (4 μL)	10^−8^ M	Not applicable	3	[25]
Slippery liquid-infused porous surface	R6G	5 min (50 μL)	10^−18^ M	Not applicable	1	[15]
Continuous-rolling-assisted evaporation on a superhydrophobic surface	CV	9 min (50 μL)	10^−15^ M	Feasible	1	[18]
Superhydrophobic magnetically functionalized PDMS	R6G	180 min (20 μL)	10^−17^ M	Feasible	9	[16,17]
Superhydrophobic concave dome array	CV	40 min (13 μL)	10^−11^ M	Feasible	36 (can be scalable)	This work

## Data Availability

Not applicable.

## Supplementary Materials

The following supporting information can be downloaded at: https://www.mdpi.com/article/10.3390/bios12050270/s1, Figure S1: On the same SCDA, different kinds and concentrations of analytes are made into multiple hot-spots for SERS detection using the enrichment mode of dynamic evaporation.; Figure S2: Images of experimental setup.; Figure S3: Raman spectrum of a 10 μL CV droplet (10^−3^ M). Video S1: The SCDA shows the excellent superhydrophobic property.; Video S2: The concave dome can physically confine the droplets.; Video S3: The analytes rotate periodically on the SCDA.
