# Supplementary material for: Effective Enrichment of Plasmonic Hotspots for SERS by Spinning Droplets on a Slippery Concave Dome Array
Source: Biosensors (Basel). 2022 Apr 24;12(5):270. doi: 10.3390/bios12050270 (PMC9138491; doi:10.3390/bios12050270)
Supplement: Supplementary file 1 [file biosensors-12-00270-s001.zip › biosensors-1681193-SI.pdf]

biosensors

Supplementary

Effective Enrichment of Plasmonic Hotspots for SERS by Spinning Droplets on a Slippery Concave Dome Array

Jialin Wu [1], Jianpeng Cai [1,2], Yuan Fan [1,2], Ying Zhang [1,2], Hui Fang [1,*] and Sheng Yan [3,*]

1 Nanophotonics Research Center, Institute of Microscale Optoelectronics, Shenzhen University, Shenzhen 518060, China; 2070496010@email.szu.edu.cn
2 College of Physics and Optoelectronics Engineering, Shenzhen University, Shenzhen 518060, China; 1900453026@email.szu.edu.cn (J.C.); 2176285320@email.szu.edu.cn (Y.F.); 1950453026@email.szu.edu.cn (Y.Z.)
3 Institute for Advanced Study, Shenzhen University, Shenzhen 518060, China
* Correspondence: fhui79@szu.edu.cn (H.F.); shengyan@szu.edu.cn (S.Y.)

Citation: Wu, J.; Cai, J.; Fan, Y.; Zhang, Y.; Fang, H.; Yan, S. Effective Enrichment of Plasmonic Hotspots for SERS by Spinning Droplets on a Slippery Concave Dome Array. *Biosensors* **2022**, *12*, 270. https://doi.org/10.3390/bios12050270

Received: 30 March 2022
Accepted: 22 April 2022
Published: 24 April 2022

Publisher's Note: MDPI stays neutral with regard to jurisdictional claims in published maps and institutional affiliations.

Copyright: © 2022 by the authors. Licensee MDPI, Basel, Switzerland. This article is an open access article distributed under the terms and conditions of the Creative Commons Attribution (CC BY) license (https://creativecommons.org/licenses/by/4.0/).

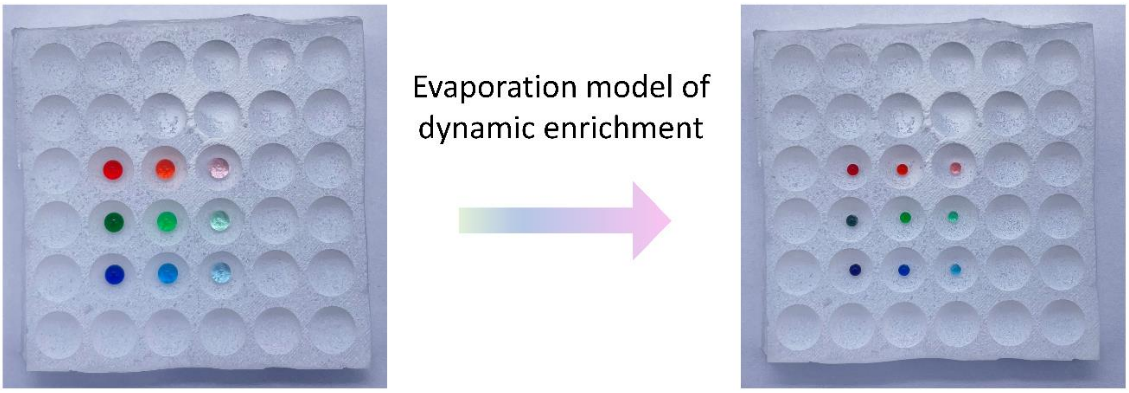

Figure S1. On the same SCDA, different kinds and concentrations of analytes are made into multiple hotspots for SERS detection using the enrichment mode of dynamic evaporation.

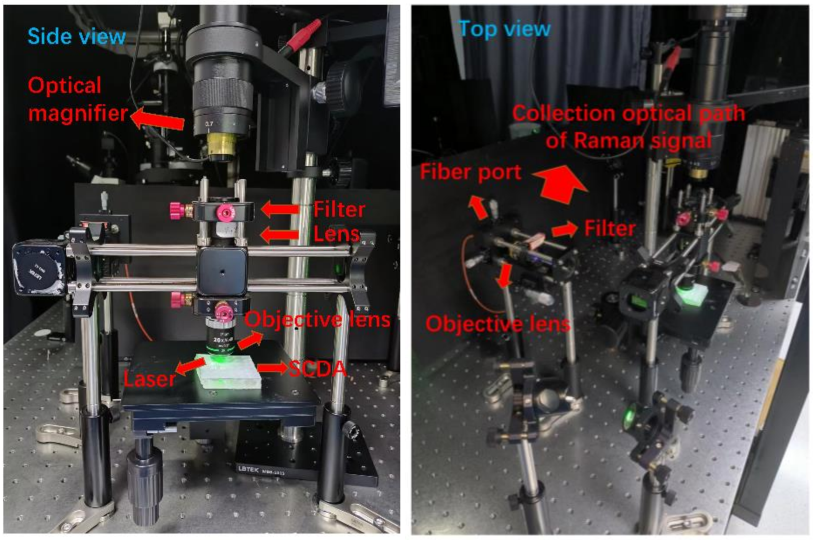

Figure S2. Images of experimental setup.

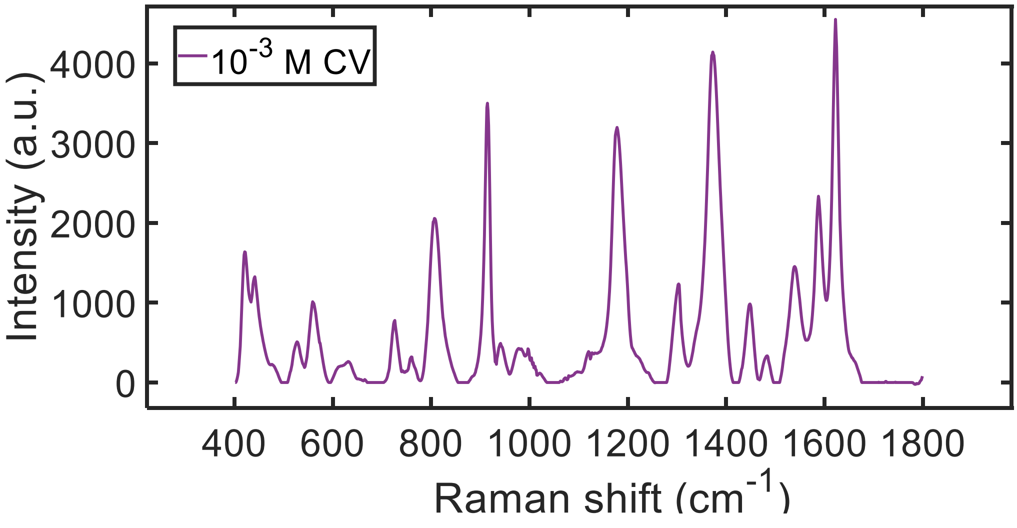

Figure S3. Raman spectrum of a 10 μL CV droplet (10^{-3} M).
